# Optimizing Transplant Approaches and Post-Transplant Strategies for Patients With Acute Myeloid Leukemia

**DOI:** 10.3389/fonc.2021.666091

**Published:** 2021-04-15

**Authors:** Justin Loke, Hrushikesh Vyas, Charles Craddock

**Affiliations:** ^1^ Centre for Clinical Haematology, Queen Elizabeth Hospital, Birmingham, United Kingdom; ^2^ Cancer Research UK Clinical Trials Unit, University of Birmingham, Birmingham, United Kingdom

**Keywords:** acute myeloid leukemia, allogeneic stem cell transplantation, graft-vs-host disease, graft-vs-leukemia, chemotherapy, MRD (measurable residual disease)

## Abstract

Acute Myeloid Leukemia (AML) is the commonest indication for allogeneic stem cell transplantation (allo-SCT) worldwide. The increasingly important role of allo-SCT in the management of AML has been underpinned by two important advances. Firstly, improvements in disease risk stratification utilizing genetic and Measurable Residual Disease (MRD) technologies permit ever more accurate identification of allo-mandatory patients who are at high risk of relapse if treated by chemotherapy alone. Secondly, increased donor availability coupled with the advent of reduced intensity conditioning (RIC) regimens has substantially expanded transplant access for patients with high risk AML In patients allografted for AML disease relapse continues to represent the commonest cause of transplant failure and the development of novel strategies with the potential to reduce disease recurrence represents a major unmet need.

## Introduction

Notwithstanding the advent of a number of novel chemotherapeutic agents for the treatment of Acute Myeloid Leukemia (AML) (1–3), allogeneic stem cell transplantation (allo-SCT) remains centrally important in the optimal management of fit adults with AML (4). Importantly, allo-SCT, consequent upon both dose intensification and the genesis of a potent donor-derived graft-vs-leukemia (GvL) effect still represents the most effective anti-leukemic therapy in adults with AML.

The last decade has seen a number of notable advances in the rationale use of allo-SCT in AML. Refinements in risk stratification of patients consequent upon the use of next-generation sequencing (NGS) (5), measurable residual disease (MRD) (6) and improvements in supportive care strategies (7) has led to more precise identification of patients who are likely to benefit from allo-SCT. At the same time the demonstration that reduced intensity conditioning regimens secure durable stem cell engraftment with a substantially lower toxicity has dramatically increased the upper age limit for allo-SCT and fit adults with AML up to the age of 75 can now be considered transplant candidates. Although a number of landmark studies of conditioning regimens have been performed in adult AML much remains to be done to personalize the transplant strategy according to both underlying disease biology and patient age and co-morbidities (8–11). At the same time the expansion of donor registries allowing increasing use of alternative donors as well as the advances in haploidentical transplantation have resulted in the great majority of allo-mandatory patients being able to access a donor (12, 13). Together these developments have cemented the position of AML as the leading indication for allo-SCT today (14).

Disease relapse however remains the commonest form of transplant failure in adults allografted for AML. Given the dismal outcome of patients with relapsed disease the development of pre-, peri- and post-transplant strategies with the potential to reduce the risk of disease relapse is now a priority and is essential if we are to improve transplant outcomes (15–17). Developments under investigation to reduce the risk of relapse post-transplant, include targeting pre-transplant MRD, optimizing the conditioning regimen and the use of maintenance therapy (18–21). At the same time the opportunity to combine either pharmacological or cellular interventions in the form of donor lymphocyte infusions (DLI) in patients with emergent disease remains an important alternative strategy to improve transplant outcomes.

## Which Patients With AML Should Be Transplanted in First Remission?

Patients with AML who relapse often fail to achieve a second remission (22) and outcomes for patients allografted in CR2 as opposed to CR1 are unsatisfactory (23). Reasons for this include both differences in disease biology at relapse which reflects selection of chemo-resistant clones (24, 25) and reduction in patient fitness due to repeated treatment. It is therefore important, where possible, to ensure the identification of patients with AML likely to benefit from an allograft whilst they are in CR1. Deciding which patients with AML in CR1 should proceed to allograft is a dynamic process driven by a calculation of i) the relative risk of disease relapse if the patients receive intensive chemotherapy (IC) alone and ii) the predicted transplant related mortality (TRM) (**Table 1**). Over time, this decision process has evolved consequent upon both improved risk stratification for CR1 patients and significant reductions in TRM.

**Table 1:** A table to demonstrate selection of patients for allogenic-SCT (Allo-SCT) with estimated relapse risk [Dohner et al. (5); Schuurhuis et al. (6)] with and without transplant and estimate of incidence of non-relapse mortality (NRM) following allo-SCT [Sorror et al. (26)].

2017 ELN risk stratification	Estimated risk of relapse following consolidation with			Maximal tolerated NRM prognostic scores for allo-SCT to be considered	
	MRD after cycle 2 chemotherapy	Chemotherapy alone (%)	Allo- SCT (%)	HCT-CI score	NRM risk (%)
Favorable	Negative	30	15-20	N/A (not advisable to proceed)	
	Positive	75	30-40	3-4	<30
Intermediate	Negative	55	25-30	2	<20
	Positive	75	35	3-4	<30
Adverse	N/A	>90	50	5	<35

Adapted from Cornelissen and Blaise 2016 (27).

Pivotal studies by the HOVON group have demonstrated that the risk of relapse in patients allografted for AML in CR1 is more than halved in comparison with those who receive intensive chemotherapy alone and that this striking reduction is consistently observed in all cytogenetic subgroups (28–30). Allo-SCT should therefore be considered recommended in patients in whom the reduction in relapse risk delivered by an allograft offsets the attendant TRM of allo-SCT. According to the ELN guideline allo-SCT should be considered in fit adults with AML whose risk of relapse if treated with intensive chemotherapy alone is more than 40% providing a suitable donor is available (5, 31). In other words, for patients who are classified as “favorable risk” according to the 2017 ELN criteria, if their risk of AML relapse is less than 40%, they should be treated with 3 to 4 courses of intensive chemotherapy and not be considered for an allograft. In contrast, allo-SCT has the potential to improve long term outcome in patients whose risk of relapse is higher than 40% (**Table 1**).

### Optimizing Risk Stratification for Patients With AML Treated With Intensive Chemotherapy Alone

Risk stratification for patients with AML can be based on a combination of clinical, molecular and response assessment to treatment. The advent of NGS mutation subtyping and measurable residual disease (MRD) monitoring in adults with AML in CR1 have led to substantial advances in risk stratification.

#### Clinical Variables Identifiable at Presentation

Clinical factors predicting an inferior outcome in a patient with newly diagnosed AML include patient age, white cell count and the presence of secondary AML (32). Despite the availability of molecular and MRD assays to refine risk assessment these risk factors remain important in determining the risk of disease relapse (26) and who might benefit from an allo-SCT.

#### Cytogenetic and Molecular Genetics Variables

Karyotypic abnormalities are present in up to 60% of AML cases and permit classification of patients into populations with a favorable, intermediate and adverse overall survival if treated with intensive chemotherapy alone (33, 34). A large proportion of AML patients either fall into the intermediate risk group following cytogenetic analysis or have no cytogenetic aberration by conventional karyotyping. Molecular analysis has increasingly identified genetic mutations with biological and prognostic significance and importantly has transformed risk stratification for patients with no detectable cytogenetic abnormality.

In the last decade the presence of mutations in transcription factors, epigenetic modifiers, spliceosome and cohesin complexes have been integrated into the risk stratification of these patients through the widely adopted European leukaemiaNet (ELN) classification (4, 5, 32, 35). The ELN 2017 classification now utilizes both karyotypic abnormalities and the presence of mutations in *NPM1, FLT3, CEBPA, RUNX1, ASXL1* and *TP53* and this risk stratification has been validated in cohorts, primarily of younger patients with AML treated with intensive chemotherapy. The widespread adoption of NGS technology permits the routine identification of a range of additional prognostic mutations in AML and is likely to inform future risk stratification models. At the same time much work remains to be done in both determining the significance of such scoring systems in older adults treated with intensive chemotherapy and understanding the prognostic significance of distinct mutational signatures (36).

#### Measurable Residual Disease

**Figure 1:**
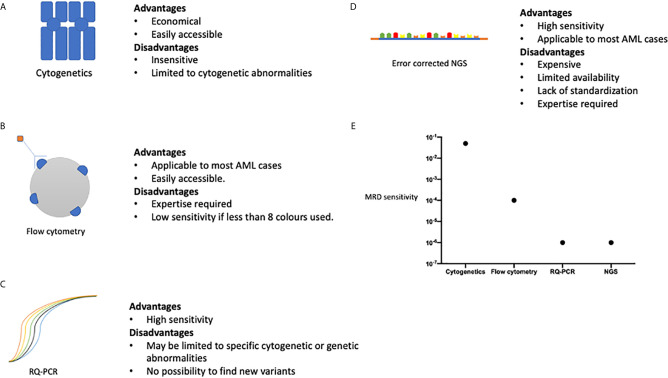
A summary of the advantages and disadvantages of methods of MRD assessment, **(A)** cytogenetic analysis, **(B)** multi-parametric flow cytometry, **(C)** Quantitative polymerase chain reaction. **(D)** Next generation sequencing. **(E)** Represents the sensitivity of each method of MRD assessment method comparing the ability of each method to detect a single AML cell amongst normal haemopoietic cells. Sensitivities are as follows: Cytogenetics 1 in 20, Flow cytometry 1 in 10,000, RQ-PCR 1 in 1,000,000, NGS 1 in 1,000,000. Data adapted from Ravandi et al. (37).

In patients who have achieved a morphological CR quantitation of MRD provides an additional prognostic tool (**Figure 1**). Sensitive measurement of the MRD load can be achieved either using RQ-PCR, NGS methodology or multi-parametric flow cytometry (MFC) designed to detect MRD residual cells with a leukemia associated immunophenotype (LAIP) (31, 34).

With the variety of methods available to quantify MRD, the question arises as to which is the best modality to use and how useful the information obtained will be in patient management? The ELN has produced guidance on the integration of MRD status in the prognostication of patients and use in clinical trials (38). MFC methodologies have the advantage of being widely available and applicable to most cases of AML, but is less sensitive than RQPCR based technologies, at approximately 1 in 10000 (**Figure 1**) (39). The ELN suggest the use of 0.1% to distinguish between MFC MRD positive and negative status, derived from patient outcomes following induction chemotherapy, but values below this may still represent residual disease (40). There are differing analytic approaches in MFC MRD in AML. One method is to identify a leukemia-associated immunophenotype (LAIP) at diagnosis and track these changes through treatment, whereas another approach is to track leukemic cells by identifying aberrant differentiation and maturation profiles [Different from Normal (DFN)]. An approach adopted by many labs is to integrate the two techniques, but there is disagreement as to the precise composition of the panels of antibody markers to identify different cell markers. As such, it is at present difficult to standardize MFC MRD between centers but attempts to develop unsupervised methodology may circumvent this limitation.

RQ-PCR based techniques have the ability to detect MRD at even lower thresholds, up to 1 in 1,000,000 cells. RQ-PCR assessment may be restricted to certain gene rearrangements and mutations; and is sometimes limited by novel breakpoints, especially in more infrequently occurring translocations. NGS is an exciting prospect allowing for the assessment of multiple gene loci and mutations in a single experimental run. However, the sensitivity of NGS is limited by its background error rate and a consensus on the methods available to correct some errors are still developing (41). In addition, consideration must be given to the targets analyzed by NGS, as although it provides the ability to look for a wide range of genetic variants in patient samples, the key is the ability to differentiate those mutations which have greater sensitivity for an impending relapse. For example, mutations found in clonal hematopoiesis of indeterminant potential (CHIP) may overlap with those in AML, but the relevance of their detection in the context of MRD is uncertain (42). Some of the limitations to the use of NGS in MRD are summarized in **Figure 1**. Error-corrected NGS MRD assays is at present prohibitively expensive in large cohorts of patients, and currently not available outside of an academic study, but has the potential to be widely adopted in the future due to the wide range of target mutations which can be monitored alongside the high sensitivity of the assay (43). Head-to-head comparisons of different MRD modalities, will be necessary to see which method will be of optimal use to patients, either individually or in combination. For example, a large study from the HOVON group demonstrated the additive prognostic benefit of MFC MRD with NGS based MRD methodology (42).

The use of MRD status may be integrated with ELN stratification to more accurately identify patients who may benefit from stem cell transplant. Importantly for patients who fall into the ELN 2017 favorable risk group, who would typically not be offered an allograft therapy, may have a predicted risk of relapse up to 70% on the basis of MRD results (37, 44, 45). The benefit of allografting younger adults with NPM1+ AML who are MRD + after two courses of induction chemotherapy following induction chemotherapy patients has been confirmed in a recent ALFA Group study (46). RQ-PCR based MRD analysis of RUNX1-RUNX1T1 transcripts can also provide accurate discernment of relapse risk in otherwise favorable risk AML (47). Finally, RQ-PCR monitoring of AML transcripts can be used to detect molecular relapse which is a pre-cursor to hematological relapse (48). For example, in the case of mutated NPM1 AML, pre-emptive therapy can be delivered prior to an allogeneic stem cell transplant (49, 50). In younger patients with intermediate risk disease who lack a detectable molecular marker the presence of MFC MRD positivity assists with risk stratification and further supports a decision to proceed to allo-SCT in CR1 (51).

### Advances in Predicting Transplant Related Mortality

It has long been known that patient fitness is an important determinant of transplant morbidity and mortality and yet assessing a patient’s fitness pre-transplant remains a challenge-particularly in older patients with AML. A thorough clinical assessment at the “bedside” still remains of paramount importance but can now be complemented by a number of comorbidity scoring systems to inform decision making. The Hematopoietic Cell Transplantation Comorbidity Index (HCT-CI) predicts TRM in patients undergoing an allo-SCT based on their comorbidities and has been validated in many large and independent cohorts (52) including a large cohort of patients with AML (53). It has more recently been adapted for mismatched donors (Augmented HCT-CI) (54). A HCT-CI score of 3 or more is associated with an increased risk of TRM and has been shown to provide better predictability when age is also taken into account (26). An alternative TRM assessment tool is the EBMT scoring system which was first devised to assess outcome of allogenic stem cell transplant for patients with chronic myeloid leukemia (55), but is now not widely used in assessing patients with AML (56). Whilst the EBMT scoring and HCT-CI take different parameters into account neither perform particularly well in older patients destined for RIC allografts where studies have shown an excess TRM in patients with an HCT-CI>1 (57). Attempts to combine the two methods to develop a compounded scoring system has been shown to improve TRM prediction (55, 56). Perhaps the most crucial limitation of any TRM scoring system is they do not account for the varying weight of a risk factor dependent on other transplant variables: for example, recipient CMV serostatus positivity are a particularly poor prognostic variable in 9/10 HLA matched donor allografts (58). Hence, the TRM estimate for any patients requires a personalized score based on patient, donor, disease and other transplant variables such as conditioning selection and GVHD prophylaxis strategy. The imprecision of any TRM prediction system is reflected by the fact that every transplant physician can recall a score of patients with a worryingly high HCT-CI- who sailed through their subsequent allograft.

Therefore, the aim of future models will be to combine such risk factors to provide a more personalized predictive scores to guide therapy, similar to what has been developed in myeloproliferative neoplasms (59). One way in which this may be possible is to improve the design of predictive algorithms through the use of artificial intelligence (60). These may allow for the advent of more complex algorithms with dynamic variables to account for the variable interaction between composite risk factors. For example, CMV seems to have a more profound negative impact on patients with low-risk disease than those with high-risk disease (56). However, the dynamic nature of these risks is illustrated by the use of Letermovir in CMV seropositive recipients (61), which may modify the impact of CMV infection on TRM to a hitherto undetermined extent.

#### Identifying the Optimal Stem Cell Source

Identifying the optimal stem cell source is both a prerequisite to proceeding with an allo-SCT, and a major step in minimizing TRM. HLA matching at HLA-A, B, C and DRB1 (62) and in European centers at DQB1as well, resulting in an 8/8 or 10/10 HLA matched donor is the current standard of care in identifying either sibling or volunteer unrelated donors. Single HLA mismatches at these loci provide inferior outcomes, especially if recipients are CMV seropositive, with progressive decrements in outcomes with an increasing number of mismatches beyond 7/8 or 9/10 HLA matches (63, 64). With high resolution HLA typing, an HLA 10/10 matched unrelated donor provided similar outcomes to those of an HLA matched sibling donor (65). Indeed, with the increasing age of patients with AML being considered for an allograft and by extension their sibling, a younger well matched unrelated donor may be preferable for reasons of donor health and data suggesting donor age may affect recipient overall survival, in the context of unrelated donors (66, 67). Similarly, in patients with multiple donors available, a number of rigorously performed studies suggests other factors may become important to consider (58, 62). Matching or permissive mismatching as predicted by T cell epitope prediction at HLA-DPB1 in recipient: donor (68) may lower the risk of non-relapse mortality. Increasing research into the impact of donor clonal hematopoiesis on recipients of an allo-SCT is likely to provide a further variable to consider for patients with more than one well matched stem cell donor (69).

Although the likelihood of identifying a HLA 10/10 matched unrelated donor for a Caucasian individual is over 75% (12), a substantial number of patients with high risk AML may still lack a HLA 10/10 or 9/10 matched donor, notably those from ethnic minority backgrounds. For these individuals, a haploidentical or umbilical cord stem cell source is a donor source with acceptable outcomes (70–72). Given the results of transplantation with these alternative stem cell sources, it is unusual not to be able to identify a suitable donor for a patient with high-risk AML. Thereby, placing greater onus on the need to start donor searches at an early stage in patients’ treatment pathway.

Whilst a number of retrospective studies have attempted to answer which alternative donor source is superior to the other a recent BMT CTN 1101 study was able to randomized patients to either an umbilical cord stem cell source or a haploidentical donor (73). The study closed prematurely due to slow recruitment, which may also affect the applicability of the results to contemporary practice. However, despite this, this important study has shown superior transplant related mortality in patients transplanted with a haploidentical donor as compared to those with an umbilical cord source, without a significant difference in relapse rates. This is in keeping with a large registry study which shows the superior outcomes in patients undergoing a RIC transplant with a haploidentical donor, in comparison with other alternative donors (74).

## Transplantation of Patients With AML Not in CR1

Patients with AML who do not achieve a remission after two cycles of induction chemotherapy are deemed to have primary refractory AML (38). The UK NCRI group recently studied more than 8,000 patients to compare the outcomes of patients with varying definitions of primary refractory disease (75). Regardless of the definitions of refractory disease, patients achieved long term survival rates in the region of 25-30% after allo-SCT. This is in concordance with other data that suggest long term survival following an allo-SCT is possible for patients with this aggressive sub-type of AML (76). Early identification of patients with primary refractory disease who may benefit from allo-SCT would improve the care of this patient group. For those considered fit for a myeloablative regimen, this is probably the optimal conditioning for their allo-SCT. In patients considered not fit for a myeloablative approach, the optimal conditioning regimen for patients with primary refractory AML has not been defined but optimism has been placed in adding (FLAMSA) sequential chemotherapy in a RIC regimen. For patients transplanted in CR2 and beyond, historical data confirms the role of allo-SCT (77) in providing a potentially curative pathway.

## Approaches to Reduce Risk of Relapse Post Stem Cell Transplant

**Figure 2:**
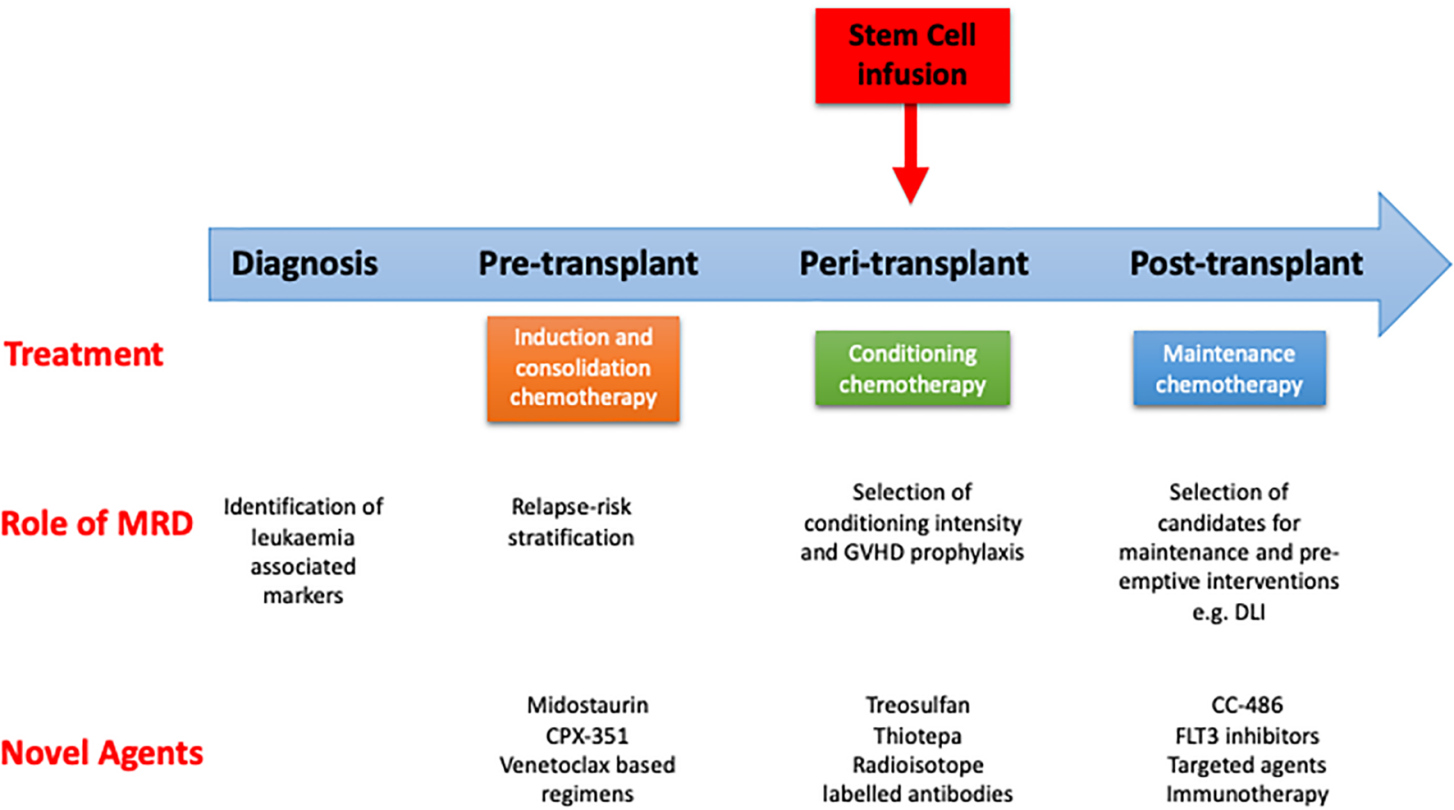
Pathway of patient with AML undergoing curative treatment. Role of measurable residual disease (MRD) and novel agents at different stages. GVHD, graft vs host disease; DLI, donor lymphocyte infusion.

Although there have been improvements in supportive care, the relapse rates for patients transplanted in CR1 remains unacceptably high at 30%-70% at 2 years (8, 10, 78). Following relapse, prognosis for patients is generally poor, especially amongst patients who relapse early post-transplant. The 2-year survival for these patients remains in the region of 20% (79). The risk factors for relapse reflect disease biology and include the presence of FLT3-ITD (80), *TP53* mutations (81), and high risk cytogenetic abnormalities (78). Disease status entering an allo-SCT is one of the most important risk factors for relapse and overall survival, with historically poor outcomes identified in those entering transplant with active disease (82, 83). In more recent years, the assessment of pre-transplant MRD has shown outcomes in some studies where the risk of relapse post-transplant with pre-transplant MRD is high, and approaches that of those entering the transplant without a morphological remission (84). Relapse is higher in patients transplanted using a RIC regimen (85). Finally, the presence of MRD early post-transplant, regardless of methodology, is a potent risk factor for relapse (86, 87). Hence, approaches to reduce the risk of relapse can be roughly divided into pre-, peri- and post-transplant factors (**Figure 2**).

### Optimizing Pre-Transplant Factors

#### What Is the Importance of Pre-Transplant MRD in Determining Transplant Outcome?

A number of retrospective studies have demonstrated that MRD status pre-transplant is a strong predictor of disease relapse post-transplant although the effect size has been highly variable. Indeed, one study identified that the risk of relapse post-transplant in patients with AML in CR1 who have detectable pre-transplant MRD is comparable to that observed in patients with active disease (84). A meta-analysis of 19 retrospective studies confirmed the predictive value of MRD status in terms of relapse risk and overall survival in patients allografted for AML (40) but until recently no prospective studies had addressed this critical question. The first prospective study demonstrated the predictive impact of pre-transplant MRD as measured by error corrected NGS in patients with AML (43). More recently the FIGARO study represented the first prospective evaluation of the prognostic impact of flow determined pre-transplant MRD in patients with AML and MDS. This study confirmed the increased risk of relapse in patients with pre-transplant MRD levels of 0.2% or above (11). Importantly, despite the increase risk of relapse in patients with higher levels of MRD, approximately 50% of patients survived at 2 years and therefore still stood to benefit from receiving a RIC allo-SCT, confirming the continued importance of allo-SCT in this sizeable patient population. Pre-transplant MRD measured by other modalities such as RT-PCR or error corrected NGS has shown this has similar important prognostic value (43, 46). For example, in patients with NPM1 mutant AML, pre-transplant MRD positivity was associated with a poorer outcome post-transplant (49). Despite this, patients with a poor NPM1 MRD response to chemotherapy stood to benefit from allo-SCT as consolidation strategy (46). Given the prognostic significance of pre-transplant MRD there is now an urgent need to prospectively evaluate whether there is any benefit of interventions designed to reduce the MRD load pre-transplant (40) (**Figure 2**). A number of options exist including the use of novel formulations of standard chemotherapy, such as CPX-351, or agents such as venetoclax, both of which merit examination in a prospective randomized control trial.

#### Can Pre-Transplant Therapy Be Optimized to Reduce Pre-Transplant MRD?

There has been no prospective study that has examined whether treatment modification pre-allo-SCT has any impact in a) reducing pre-transplant MRD levels, or, b) increasing overall survival post allo-SCT. Firstly, there is no consensus on the optimum number of cycles of induction chemotherapy prior to allo-SCT. Retrospective studies looking at the impact of additional consolidation chemotherapy with cytarabine following remission induction in patients undergoing reduced intensity or myeloablative conditioning failed to show any significant benefit in terms of overall survival or disease relapse (39, 88). Prospective studies in this area are lacking and so we do not know whether there is any benefit or harm from additional chemotherapy cycles following induction. However, data from patients with primary refractory AML suggest that additional courses of chemotherapy in patients with chemo-resistant disease is detrimental (75).

In recent years, a number of novel agents as part of induction and consolidation strategies have received FDA approval (2, 89, 90), and provide some provocative, preliminary, data to suggest that optimizing pre-transplant chemotherapy has an impact on post-transplant outcomes. The use of midostaurin as an adjunct to conventional chemotherapy in induction, consolidation and maintenance phase has demonstrated overall survival benefit in a randomized controlled trial (2) in patients with FLT3 mutant AML. From these patients, approximately 55-60% underwent allo-SCT and in this subset of patients a trend to benefit was retained in those receiving midostaurin as compared to control (2). Novel FLT3 inhibitors such as Gilteritinib and Quizartinib provide a route for patients with relapsed FLT3 mutant AML to achieve remission and to further consolidation with an allo-SCT (91, 92).

Similarly, in a trial comparing CPX-351 to conventional DA 3 + 7, benefit in terms of overall survival and remission rates in patients in the CPX-351 arm was seen but interestingly this significant survival benefit was retained when comparing patients in the CPX-351 arm compared to standard therapy in patients who subsequently underwent an allo-SCT (93). As there could be potentially a number of mechanisms behind the survival advantage of these novel agents in the cohort of patients who have received an allo-SCT, it will be important in future studies to incorporate pre-transplant MRD to ascertain if this advantage is a result of a deeper remission status. Hence, there is a need for further adequately powered, randomized, prospective trials to assess if these agents improve pre-transplant MRD and reduce risk of relapse post-allo-SCT. It is of particular interest that an ongoing randomized control trial investigating the benefits of CPX-351 vs intermediate dose cytarabine at consolidation, will assess the impact of this cycle of treatment on MRD pre-transplant, and subsequent post-transplant outcome (COSI, NCT04217278).

The improvements in remission rates and survival in patients receiving venetoclax in addition to azacitidine (94) has increased the number of patients being considered for an RIC allo-SCT who otherwise may not have attained a remission to reach this treatment stage. It remains to be seen whether the depth and quality of remissions achieved in patients with venetoclax based combinations are comparable to patients receiving conventional intensive chemotherapy prior to an allo-SCT, and whether, this has any long-term significance in their overall survival. The use of venetoclax based regimens may enable patients to reach an allo-SCT with a reduction in morbidity. This is similar to the use of azacitidine to achieve remission, prior to allo-SCT, in elderly patients (95).

### Optimizing Peri-Transplant Factors for Patients With AML

#### Historical Development of Conditioning Regimens for Patients With AML

The role of the conditioning regimen in allo-SCT in patients with AML is to allow durable engraftment of the donor stem cells and to deliver a direct cytotoxic anti-leukemic effect. This was illustrated by early observations, in patients with AML, that conditioning intensity correlated with relapse risk (96). Myeloablative regimens (MAC) arose from the origins of hematopoietic stem cell transplantation (97) and such regimens are defined by the induction of permanent bone marrow aplasia, in the absence of hematopoietic stem cells infusion. Important studies to support the benefits of allo-SCT in patients undergoing myeloablative sibling allografts for AML in CR1 compared the outcomes of patients with AML who had an HLA-matched sibling donor for allo-SCT, or not (28) (“donor vs no-donor” methodology). The benefits of such intensive regimens were only seen in patients under 40 years of age, due to an excess in TRM (28) in the patients older than 40 years of age.

MAC regimens historically combined Cyclophosphamide (Cy) with either TBI or busulfan (Bu) and remain in common use. However, new developments in conditioning regimens have extended the applicability of MAC regimens. Critically, intravenous preparations of busulfan have led to more predictable pharmacokinetic properties and better tolerability such that they are at least equitable to TBI based regimens. Indeed, one prospective study suggested a superior overall survival in patients treated with a Bu- as opposed to a TBI- based regimen (98). This randomized control trial (RCT) demonstrated a marked improvement in NRM for Fludarabine (Flu)/Bu4 (12.8 mg/kg over 4 days of IV busulfan) over an iv-Bu/Cy regimen (same dose of busulfan), in a population of patients aged 40-65. As such Flu/Bu4 is now widely used as a standard of care regimen for patients receiving an allo-SCT as a MAC protocol.

Reduced intensity conditioned (RIC) regimens have played an important role in extending the age at which adults with high risk AML can be safely allografted. A RIC regimen is defined as incorporating ≤8 Gy Total Body Irradiation (TBI) or ≤8 mg/kg busulfan (99) and a number of variously myelosuppresive and immunosuppressive iterations exist. Taken together however the advent of RIC regimens has dramatically extended the spectrum of patients with AML who may be a candidate for a RIC allo-SCT, such that fit patients with AML in remission over the age of 75 are now routinely transplanted in many transplant centers with acceptable results (100, 101). The optimal RIC regimen has not yet been established and is likely to depend on both underlying disease biology, patient age and donor source. One of the first randomized studies in AML compared outcomes in patients allografted with a Flu/Bu2 (6.4 mg/kg, two days of IV busulfan) RIC regimen and a non-myeloablative Flu/2Gy TBI regimen, Despite a similar overall survival in both groups, there were significant differences in TRM and relapse rates with a markedly higher relapse rate in the Flu/2GyTBI regimen (102). A more recent study compared the Flu/Bu2 regimen with a Flu/Treosulfan (10g/m^2^ for 3 days) regimen This demonstrated non-inferiority between the two regimens, with similar relapse rates, but a reduction in TRM for patients treated on Flu/Treosulfan arm (103), although interpretation of this study is hampered by the unusually high TRM seen in the Flu/Bu2 arm of the study. In an attempt to reduce relapse rates post-transplant, which remains the major cause of treatment failure after a RIC allo-SCT, the addition of sequential chemotherapy to transplant conditioning has been compared to standard RIC regimens. The FIGARO study demonstrated that the FLAMSA-Bu regimen did not improved survival or relapse risk as compared to a standard RIC regimen in patients with high risk AML or MDS (11).

#### Should the Presence of Pre-Transplant MRD in Patients With AML in CR1 Influence the Intensity of the Conditioning Regimen?

A number of randomized trials have sought to determine whether a RIC or MAC regimen is preferable in patients with AML in CR1 aged between 40-60 with AML or MDS. To date three studies have directly addressed this important question (9, 10, 104, 105). However, as a result of the enduring challenge of achieving timely recruitment to randomized transplant trials two were underpowered which complicates their interpretation. Of note two studies have demonstrated similar outcome with either AML or MDS transplanted using a MAC or RIC regimen (104, 105). In contrast a more recent US BMT CTN (0901) study which recruited briskly and randomized patients to either a RIC or MAC regimen showed a higher rates of relapse in patients transplanted using a RIC regimen. Although this resulted in an inferior RFS there was no statistically significant difference in survival between the two arms (9) in the original report. A recent update with long term follow up, suggest that there is a superior OS in the MAC as compared to the RIC arms of the study (106). Interpretation of this important trial is however complicated by the unexplained discrepancies between the relapse rates observed in both RIC AND MAC arms compared with those observed in other studies.

An important further consideration is whether the choice of conditioning regimen should be determined by pre-transplant MRD status. In important adjunctive studies of the BMT CTN 0901 cohort Hourigan demonstrated improved survival in MRD+ patients with AML who were transplanted using a MAC as opposed to a RIC regimen. Of interest the outcomes of MRD- patients who received either a MAC or RIC was equivalent. In contrast no clinical benefit was observed in MRD + patients transplanted using the intensified sequential FLAMSA regimen in the FIGARO trial (11).

Further studies are therefore required in order to robustly examine the optimal conditioning regimen in patients with detectable MRD pre-transplant (43). Due to the likely beneficial impact of myeloablative conditioning on relapse risk, patients should be assessed on an individual basis to determine the likely benefits of a more intensive conditioning regimen. A RIC regimen is to be preferred in older patients in whom the TRM of a MAC regimen is deemed likely to be excessive. In the sizeable population of older patients who will, *force majeur*, be transplanted using a RIC regimen post-transplant strategies aimed at maximizing a GVL effect should be prioritized.

#### Can We Increase the Anti-Leukemic Properties of Conditioning Regimens Without Excess Toxicity?

Thiotepa is an alkylating agent that has been used in a number of transplant conditioning regimens, including a Flu/Bu/Thiotepa regimen which had been used for patients undergoing umbilical cord transplants with low relapse risks (107). In recent years promising results have been seen when thiotepa (5mg/kg/day for 2 days) has been added to Flu/Bu regimens (fludarabine 50 mg/m^2^/day for 3 days, busulfan at 3.2mg/kg/day for 3 days) (108) in reducing relapse risk in patients with AML in CR1 (109, 110). The COSI study is a randomized control trial currently undergoing recruitment and will compare the Flu/Bu4 regimen with the Flu/Bu/Thiotepa schedule in patients under 55 years of age, whilst those over 55 years will either receive a Flu/Bu2 schedule or a miniThiotepa/Bu/Flu regimen (thiotepa 5mg/kg for 1 day, fludarabine 50 mg/m^2^/day for 3 days, busulfan 3.2mg/kg/day for 2 days).

Conditioning chemotherapy may also be sequenced in novel regimens to allow older patients to have more potent treatment (111). Another strategy that remains in development is the use of radio nucleotide labelled antibodies to reduce the side effects experienced with conventional chemotherapy-based conditioning regimens. One example that is undergoing clinical trial is the 131-iodine labelled anti-CD45 antibody (112). BC8, delivered as part of conditioning alongside fludarabine and 2 Gy TBI. CD45 is a promising target due to its widespread expression on hematopoietic cells but not in non-hematopoietic tissue, thereby potentially reducing the incidence of adverse effects.

#### Optimizing GVHD Prophylaxis

Both acute and chronic GVHD are an important cause of morbidity and mortality. Hence a composite outcome measure for studies in allo-SCT has been rapidly established which combines GVHD and relapse free survival (GRFS) (113). An early observation in the history of allogeneic stem cell transplantation was that GVHD was inversely correlated with the risk of relapse, supporting the presence of a graft-vs-leukemia effect (114). This has been reinforced by later observations that the level of cyclosporine exposure through dosing, especially in the early days of transplant can significantly influence the risk of relapse (115, 116). T cell depletion (TCD) can be used to reduce the incidence of GVHD (117) and *in vivo* strategies involving anti-thymocyte globulin (ATG) or anti-CD52 antibody (alemtuzumab) are in routine use (118, 119). However, TCD as compared to T replete transplants, are associated with increased risk of disease relapse (120), viral infections (e.g. CMV) (121), and a significant incidence of mixed donor: recipient chimerism in the T cell fraction. Further developments in GVHD prophylaxis continues with increasing uptake of post-transplant cyclophosphamide, beyond the setting of haploidentical donor transplants, into patients receiving a MUD allo-SCT (122, 123), although a definitive prospective study comparing these different forms of GVHD prophylaxis remains to be seen, but are underway (124). In the future, optimizing GVHD prophylaxis may involve taking into account different factors including disease risk, donor source, and conditioning regimen.

### Optimizing Post-Transplant Strategies for Patients With AML

The risk of relapse in patients allografted for AML ranges between 30-70% and is dependent on disease biology, remission and MRD status pre-transplant and conditioning regimen intensity (29, 125) (**Figure 2**). These risk factors identify a cohort of patients with a high risk of relapse post allo-SCT in whom post-transplant strategies to reduce this risk is urgently required. A further complication in the management of this post-transplant period is the variability in post-transplant recovery and potential other complications such as infection and GVHD which will influence the feasibility of any pharmacological intervention. Therefore, post-transplant strategies to prevent relapse, as in other aspects of transplant care, should be optimized to the individual.

#### Post-Transplant Strategies to Monitor Disease

The presence of detectable MRD at early time-points post-transplant is associated with a very high risk of relapse (126) and this has been confirmed using NGS MRD technologies (86, 127). For example in t (8;21) AML, failure to achieve a 3 log reduction in transcripts, as compared to baseline pre-treatment levels, by month 3 post-transplant is associated with a 51% risk of relapse as compared to only 8% in those who achieve this landmark (128). As a consequence, there is increasing interest in routine monitoring of MRD post-transplant in patients allografted for AML. However, the complexity in interpreting post-transplant MRD results is exemplified in the context of NPM1 monitoring where post-transplant relapse risk is dependent on the level of MRD (129). Of note, not all patients with detectable MRD post-transplant proceed to overt relapse suggesting the possible role of an emergent GvL effect in disease control. However, the detection of MRD post-transplant mandates urgent consideration of interventions such as a use of donor lymphocyte infusions (DLI). Such interventions are more effective in patients with a lower disease burden (130, 131) and hence maybe more effective in molecular relapse, prior to hematological relapse (132, 133). Likewise, the experience of using azacitidine post allo-SCT with relapsed AML, blast percentage in the marrow is an independent prognostic factor for survival (16).

Donor: recipient chimerism monitoring can be accomplished routinely either by short tandem repeat (STR) or sex difference between donor and recipient using PCR. This test can be done on whole blood or specific subsets such as myeloid, T Cell or CD34+ cells (134). Although, the sensitivity of this is limited to 0.1-1% depending on methodology, falling donor chimerism is associated with an increased risk of relapse (135). The challenge in using mixed chimerism levels as a risk factor for disease relapse, is that in RIC allografts, incidences of mixed chimerism is commonly seen and is related to pharmacological levels of T cell depleting agents such as alemtuzumab (136). Chimerism can also be altered by other factors including viral reactivation, changes in immunosuppression, affecting interpretation of results (137). Fundamentally this is a result of the fact that chimerism is not a direct marker of disease.

The recent report of the FIGARO study has demonstrated the importance of interpreting pre-transplant MRD with post-transplant chimerism monitoring (11). This study demonstrated that the acquisition of full donor chimerism at 3 months mitigated the increase risk of relapse from positive pre-transplant MRD. This may provide the rationale for interventions that can increase the kinetics of attaining full donor chimerism such as modifications in immunosuppression strategies.

#### New Developments in Post-Transplant Maintenance Strategies

Post-transplant pharmacological interventions may deliver produce a direct cytotoxic effect, and augment the effects of the conditioning regimen, enabling sufficient time for the development of an allo-reactive T and B cell response against leukemic cells. However, it is also increasingly recognized that pharmacological agents may interact with the immune system to accelerate the development of a graft-vs-leukemia effect, as seen in the metabolic re-programming of leukemia reactive T-Cells in patients treated with sorafenib post-transplant (138). Development of maintenance strategies post allo-SCT are dependent on the use of agents with a) clinical activity, b) tolerable side effect profile, and which does not exacerbate cytopenias, infection rates and graft-vs-host disease. Finally, it is unclear whether maintenance strategies definitively prevent relapse or merely delays it which relates to uncertainty over the duration with which these agents should be used. Nevertheless, such strategies, even it were to delay relapse may allow the development of an effective graft vs leukemia effect through immune reconstitution.

##### Non-Targeted Agents

The use of non-mutation specific agents has the advantage that it is not susceptible to changes in clonal landscape that occurs pre- and post- transplant relapse (139, 140). Lenalidomide is effective at relapse of AML (17) in combination with azacitidine, however this may exacerbate GVHD if used as maintenance therapy alone (141). Azacitidine has been shown to be well tolerated post-transplant and may both reduce risk of GVHD through expansion of regulatory T cells and increase GvL effect by upregulating the expression of cancer testes antigens on leukemia cells (18) which is associated with a reduced risk of relapse (19). In a randomized control study, the subcutaneous administration of azacitidine, as compared to control, did not provide any additional benefits to patients with AML post allo-SCT, but this study was notable for a short on-treatment duration (142). In the RELAZA2 study, MRD measurement through donor chimerism measurement in CD34+ selected cells in post allo-SCT patients was used to select patients who would receive pre-emptive azacitidine (21). This strategy lengthened the duration of relapse free survival, in comparison to their historical experience. Much interest has been shown in the oral azacitidine formulation (CC-486), given its results in improving overall survival in elderly patients with AML who are ineligible for allo-SCT (143). In the post allo-SCT setting, CC-486 can be given with acceptable side effects (144) resulting in an ongoing phase III randomized control trial (NCT04173533). The HDAC inhibitor, Panobinostat has also been used as part of a maintenance strategy post allo-SCT in MDS or AML as part of a phase I/II study and has shown encouraging rates of GVHD as well as improved relapse and survival, leading to a phase III trial in this setting (NCT04326764).

##### Targeted Agents

A number of targeted agents designed against specific key pathways in AML has shown anti-leukemic activity in newly diagnosed and relapsed-refractory patients, resulting in a number receiving Food and Drug Administration (FDA) approval (145). Importantly many of these new agents are tolerable and can be administered in the outpatient setting, which is vital for any maintenance strategy. The hedgehog signaling pathway is important in embryonic development, and aberrant activity in this pathway is associated with chemo-resistance in pre-clinical models of AML (146). Glasdegib, is an inhibitor of the hedgehog pathway and when used with low dose cytarabine (LDAC) was superior to LDAC alone, with a short improvement in overall survival in a randomized phase II study (147). With these results the use of glasdegib as maintenance post allo-SCT in patients with high risk AML is undergoing a phase II clinical trial. IDH1 and 2 inhibitors, given its anti-leukemic activity and tolerability (148, 149) maybe a useful maintenance option for patients with IDH1/2 mutant AML.

*FLT3* is a member of the type 3 receptor tyrosine kinase family (150), mutations of which can be found in approximately 30% of patients, and is associated with increased relapse rates (151). Despite an allo-SCT, patients with FLT3-ITD mutant AML have an increased relapse rate as compared to patients with FLT3-ITD negative AML (80). Recent years have seen the clinical development of a number of FLT3 inhibitors both in frontline (2) and in the relapse-refractory setting (91, 92). First generation FLT3 inhibitors such as sorafenib, lestaurinib and midostaurin have a wider spectrum of activity against other receptor tyrosine kinases, with a broader adverse event profile and less potent monotherapy activity. However, despite this, in combination with induction chemotherapy, sorafenib improved relapse free survival (152), and in the case of midostaurin, improved overall survival. Interestingly, in the case of sorafenib, improvements in relapse free survival were irrespective of FLT3 mutation status. The RATIFY study, which allowed midostaurin to receive FDA approval was not designed to examine the role of midostaurin as post allo-SCT maintenance. Midostaurin has been used as a post allo-SCT maintenance agent in two studies. In a study of 284 patients with FLT3-ITD AML, midostaurin was used in combination with induction chemotherapy followed by allo-SCT for 12 months of maintenance (153). The results of this study were not definitive, as the outcomes could only be compared with historical controls. Similarly, a small phase II randomized control study compared midostaurin to placebo as a maintenance post allo-SCT, which showed that this agent was tolerable but the study was insufficiently powered to show statistical significance (154). Much enthusiasm for the use of sorafenib post allo-SCT was generated by the SORMAIN study (20) which although required five years for completion, and was stopped due to incomplete recruitment, demonstrated improved overall survival and reduction in relapses, albeit in small numbers. However, another randomized controlled study of Sorafenib vs control has also shown similar results to the SORMAIN study (155). Although some centers now routinely use this agent as post allo-SCT maintenance for FLT3-ITD AML, the tolerability of this agent remains debatable and questions remain over the validity of these results in the era of patients routinely receiving midostaurin at induction pre allo-SCT (156). It is in this context that second generation FLT3 inhibitors which are more specific and have potency as monotherapy, even in relapsed refractory AML such as Quizartinib and Gilteritinib (91, 92) may be important as maintenance post allo-SCT, and the results of the BMT-CTN 1506 study which compares Gilteritinib to control post allo-SCT and has completed accrual, is eagerly awaited.

### Summary

Allo-SCT now plays a central role in the management of adult AML. Yet much remains to be done improving transplant outcomes. Randomized clinical trials of novel strategies to reduce the risk of disease relapse post-transplant are now a priority. Allo-SCT provides an important platform to manipulate the immune environment against residual leukemic cells. For example, use of check-point inhibitors and other cellular therapies may become important in the future (157–159). At the same time, it is increasingly clear that integrated MRD and genomic analyses will increasingly permit adoption of personalized transplants. The effective delivery of such studies will demand a greater spirit of collaboration between clinicians and basic scientists as well as the establishment of effective national and international transplant trials networks.

## Author Contributions

All authors contributed to the writing of this review article. All authors contributed to the article and approved the submitted version.

## Funding

Research support and clinical trials funding from CRUK, Bloodwise and Cure Leukaemia acknowledged. Core funding to the Birmingham ECMC Centre program is gratefully acknowledged. The funder bodies were not involved in the study design, collection, analysis, interpretation of data, the writing of this article or the decision to submit it for publication.

## Conflict of Interest

CC has received honoraria from Celgene, Daichi-Sankyo, Novartis, and Pfizer as well as research funding from Celgene. JL has received travel funding from Novartis and Daichi-Sankyo, and honoraria from Pfizer, Janssen, and Amgen.

The remaining author declares that the research was conducted in the absence of any commercial or financial relationships that could be construed as a potential conflict of interest.
